# Rheological Behavior and Mechanical Performance of Poly(3-hydroxybutyrate-co-3-hydroxyvalerate)/Natural Rubber Blends Modified with Coffee Oil Epoxide for Sustainable Packaging Applications

**DOI:** 10.3390/polym17101324

**Published:** 2025-05-13

**Authors:** Rinky Ghosh, Xiaoying Zhao, Yael Vodovotz

**Affiliations:** 1 Department of Food Science and Technology, The Ohio State University, 2015 Fyffe Road, Columbus, OH 43210, USA; ghosh.245@osu.edu; 2 School of Light Industry Science and Engineering, Beijing Technology and Business University, No. 33 Fucheng Road, Beijing 100048, China; zhaoxy@btbu.edu.cn

**Keywords:** PHBV, NR, coffee oil epoxide, extrusion, food-waste, chemometrics, polymer miscibility, Cole–Cole plot, deconvolution, sustainable packaging

## Abstract

The inherent brittleness of bio-based poly(3-hydroxybutyrate-co-3-hydroxyvalerate) (PHBV) significantly restricts its industrial applications despite its industrial compostability. Blending with elastomeric polymers addresses mechanical limitations; however, interfacial incompatibility compromises miscibility as our previous work established. Herein, we investigate coffee oil epoxide (COE) as a bio-based plasticizer for PHBV/natural rubber (NR) blends in sustainable packaging applications. COE, derived from spent coffee grounds, was incorporated into PHBV/NR/peroxide/coagent composites via twin-screw extrusion. FTIR spectroscopy with chemometric analysis confirmed successful COE incorporation (intensified CH_2_ stretching: 2847, 2920 cm^−1^; reduced crystallinity), with PCA and PLS-DA accounting for 67.9% and 54.4% of spectral variance. COE incorporation improved optical properties (7.73% increased lightness; 21.9% reduced yellowness). Rheological characterization through Cole–Cole and Han plots demonstrated enhanced phase compatibility in the PHBV/NR/COE blends. Mechanical testing showed characteristic reductions in flexural properties: strength decreased by 16.5% and modulus by 36.8%. Dynamic mechanical analysis revealed PHBV/NR/COE blends exhibited a single relaxation transition at 32 °C versus distinct glass transition temperatures in PHBV/NR blends. Tan δ deconvolution confirmed the transformation from bimodal distribution to a single broadened peak, indicating enhanced interfacial interactions and improved miscibility. These findings demonstrated COE’s potential as a sustainable additive for biodegradable PHBV-based packaging while valorizing food waste.

## 1. Introduction

The global plastic packaging industry, predominantly reliant on conventional petroleum-derived plastics, is under increasing pressure to mitigate its environmental impact, particularly concerning persistence and waste management, particularly in consumer-facing applications like food packaging [1,2,3,4]. Food packaging represents a vital sector, contributing substantially to plastic consumption while grappling with unique sustainability challenges linked to food safety mandates, post-consumer contamination, and intricate material compositions [5,6]. The industry is actively seeking bio-based and biodegradable alternatives to enhance sustainability.

Industry projections indicate significant growth in the bioplastics sector, with production volumes expected to increase from 2.2 million tons in 2022 to approximately 6.3 million tons by 2027 [7]. This projected growth trajectory underscores the expanding market presence and technological development of biodegradable alternatives to conventional plastics.

As promising options, Poly(3-hydroxybutyrate-co-3-hydroxyvalerate (PHBV) offers significant environmental advantages as it is fully compostable and can be produced through microbial synthesis using renewable feedstocks [8]. Research demonstrates that PHBV decomposes effectively across diverse environments, including industrial and home composting settings under both aerobic and anaerobic conditions, as well as in marine ecosystems [9,10]. From a performance standpoint, PHBV demonstrates mechanical properties comparable to polypropylene, exhibiting a Young’s modulus of approximately 1.7 GPa and tensile strength of 38 MPa [11]. These characteristics establish PHBV as a potential substitute for conventional petroleum-based polymers in various applications, including rigid food packaging containers, agricultural mulch films, disposable cutlery, and biomedical devices requiring predetermined degradation profiles [11,12]. However, Meereboer et al. (2020) reported that despite its potential, PHBV currently remains less competitive compared to commodity polymers like polyethylene and polypropylene, primarily due to its high crystallinity level, inherent brittleness, and higher production costs—averaging approximately €5/kg—which collectively limit its applications in food packaging [13,14]. Additionally, the material’s high crystallinity (reaching up to 70%) and slow nucleation rate contribute to brittleness and suboptimal mechanical properties [15].

To address these limitations, numerous studies have investigated the potential of blending PHBV with flexible biopolymers to enhance its toughness and reduce crystallinity [16,17,18]. The scientific literature contains extensive research on blending PHBV with other polymers as a strategy to improve its mechanical performance [19,20].

However, compatibility between PHBV and blending polymers remains a critical challenge affecting material performance, often necessitating compatibilizers to enhance miscibility and prevent phase separation over time. Among various blending options, previous studies demonstrate that combining PHBV with natural rubber (NR) not only enhances resilience but also reduces production costs due to NR’s significantly lower price point [21]. However, these blends exhibit significant limitations during industrial thermoforming operations, primarily attributed to insufficient melt strength and two-phase morphology with weak interfacial interactions between phases [22].

External plasticization represents another approach for improving the plasticity and toughness of PHBV [23,24]. Plasticizers effectively reduce glass transition temperature and brittleness while simultaneously functioning as processing aids through melt viscosity reduction [23]. Various vegetable oils, including coffee, broccoli, soybean, castor, palm, and linseed oils, can be epoxidized for incorporation as plastic additives to enhance thermal stability, mechanical performance, and oxidative resistance [25,26]. Slongo et al. (2018) examined the plasticizing effects of dioctyl phthalate (DOP), epoxidized soybean oil (ESO), and triethyl citrate (TEC) on the thermal and mechanical properties of PHBV [27,28]. Their findings revealed that among the evaluated plasticizers, ESO demonstrated limited effectiveness in improving PHBV’s thermal and mechanical performance.

Coffee, recognized as the second most consumed beverage globally and a significant commodity, generates substantial waste in the form of spent coffee grounds after brewing, constituting approximately 90% of the original coffee bean mass [29]. Environmental concerns regarding the disposal of coffee processing waste primarily stem from the release of bioactive compounds including caffeine, tannins, and polyphenols [30]. Without appropriate management strategies, these compounds can potentially compromise ecosystem integrity due to their biological activity and persistence. The incorporation of coffee waste derivatives into polymer formulations represents a dual-benefit approach: valorizing an abundant waste stream while simultaneously developing environmentally advantageous packaging materials as alternatives to conventional petroleum-based plastics, which lack both renewable origin and biodegradability [31].

The present investigation explores the valorization potential of spent coffee grounds through innovative extraction methodologies. Williamson et al. developed a novel extraction and modification protocol to recover valuable lipid fractions from spent coffee grounds [32]. These recovered oils demonstrate potential for incorporation into PHBV-based bioplastic formulations, offering promising solutions for biodegradable food packaging applications [2,33]. This approach provides multiple sustainability benefits through effective waste stream utilization, production cost reduction, and enhanced processing efficiency in manufacturing operations [33]. Despite these preliminary studies exploring commercially available plasticizers for PHBV modifications, the existing literature lacks comprehensive analysis regarding the effects of coffee oil epoxide plasticizers on the mechanical, thermal, barrier, and morphological characteristics of PHBV/NR blends. In our previous research, we extensively studied the thermal, barrier, and morphological characteristics of PHBV/NR/COE blends [24]. Building on these findings, the present investigation systematically evaluates the impact of coffee oil epoxide, a bio-based plasticizer, on the thermal, barrier, and basic morphological properties; the current study provides comprehensive rheological characterization (Cole–Cole plots, Han plots, h-parameter analysis), mechanical behavior under different loading conditions (tensile vs. flexural), and detailed viscoelastic analysis through DMA with tan δ deconvolution. This research assesses the implications of coffee oil epoxide incorporation to develop more sustainable, effective, and environmentally benign biodegradable packaging materials with substantial commercial viability.

## 2. Experimental Section

### 2.1. Materials

Poly(3-hydroxybutyrate-co-3-hydroxyvalerate) (PHBV) granules with 2 mol% hydroxyvalerate content and a molecular weight of 280 kDa were purchased from Tianan Biological Material Co. (Ningbo, China). Trimethylolpropane triacrylate (TMPTA) and 2,5-Bis(tert-butylperoxy)-2,5-dimethylhexane (Luperox 101XL45, 92%, MW: 290.44) were obtained from Sigma-Aldrich (St. Louis, MO, USA) and Thermo Fisher Scientific, respectively. Natural rubber (NR) was supplied by Midwest Elastomers Inc. Coffee oil epoxide (COE) was prepared according to the method described by Williamson et al. (2022). Both NR and peroxide were used as received [32].

### 2.2. Melt Processing

**Figure 1:**
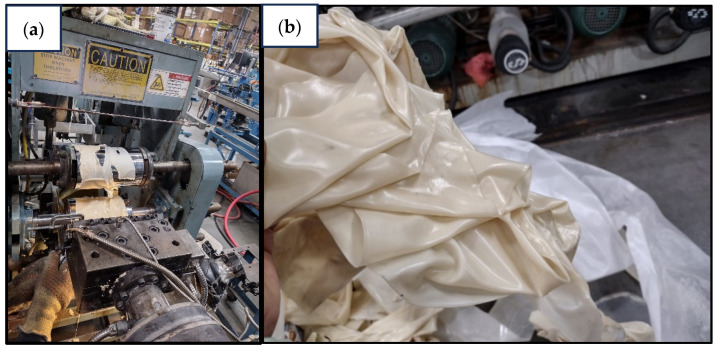
Processing challenges with higher COE concentrations (>0.5 wt%) during PHBV/NR sheet extrusion: (**a**) material adhesion to extrusion equipment demonstrating excessive stickiness and (**b**) severe sheet wrinkling and folding after extrusion, confirming the need for limiting COE content to 0.3 wt%.

Prior to processing, PHBV pellets were vacuum dried at 60 °C for 24 h, resulting in a final moisture content of 0.1% in the blends. The optimized blend formulation comprised of 85 wt% PHBV, 15 wt% NR, 0.45 wt% peroxide, 0.63 wt% coagent, and 0.3 wt% COE. The COE concentration (0.3 wt%) was selected based on our previous study (Ghosh et al. 2024), which demonstrated notable improvements in oxygen and water vapor barrier properties at this level [24]. As shown in Figure 1, sheet extrusion trials with COE concentrations above 0.5 wt% resulted in excessive surface stickiness and wrinkling, leading to sheet folding, adhesion to the rollers, and overall poor dimensional stability. This visual defect confirmed the need to limit COE content to 0.3 wt% for maintaining processability.

Melt compounding was performed using a Leistritz ZSE-27 co-rotating twin-screw extruder (D = 27 mm, L/D = 40:1) with a reverse temperature profile decreasing from 180 to 160 °C. The screw speed and throughput were maintained at 64 rpm and ~10 kg/h, respectively, with screw configuration optimized to minimize thermal degradation during processing (Table 1). The extrudate was immediately pelletized using a Scheer Bay pelletizer at a feed roller speed of 2–3 rpm.

Sheet extrusion was conducted by reprocessing the compounded pellets using an extruder equipped with a three-layer multifold die (MuCell Extrusion LLC, Woburn, MA, USA). The temperature profile was initially set at 190–202 °C and subsequently reduced by 10 °C across the extruder and die zones to enhance melt viscosity. The die gap was maintained between 0.51 and 0.89 mm throughout processing. The extruded sheets were passed through a water-cooled roll stand at ambient temperature to prevent surface adhesion and ensure dimensional stability. Processing parameters remained constant to maintain experimental reproducibility. The blends containing PHBV/NR with coagent, and peroxide were designated as PHBV/NR, while those incorporating COE were denoted as PHBV/NR/COE.

## 3. Characterization of PHBV and Its Blends

### 3.1. ATR-MIR

FTIR spectra were recorded in triplicate using a 4500 FT-IR portable spectrometer (Agilent Technologies, Santa Clara, CA, USA) equipped with a triple bounce diamond ATR crystal, quartz beam splitter, and deuterated triglycine sulfate (DTGS) detector. Measurements were performed at 4 cm^−1^ resolution over the range of 4000–700 cm^−1^, with each spectrum representing an average of 64 scans.

#### Chemometric Analysis

The acquired IR spectra were processed and analyzed using MetaboAnalyst 6.0. Data preprocessing involved normalization followed by Savitzky–Golay transformation using a 35-point window size and quadratic polynomial order to compute first and second derivatives. This preprocessing step enhanced the focus on significant spectral features. Principal Component Analysis (PCA), an unsupervised analysis, was performed to evaluate natural clustering patterns in the spectral data of PHBV and its blends. Following the unsupervised analysis, Partial Least Squares Discriminant Analysis (PLS-DA) was performed to develop refined qualitative models. Variable Importance in Projection (VIP) scores were calculated to identify the spectral regions most significant for sample discrimination.

### 3.2. Optical Properties

The optical properties of PHBV, PHBV/NR, and PHBV/NR/COE sheets were evaluated using a HunterLab ColorQuest XE MiniScan 4500 L colorimeter (Reston, VA, USA). Color measurements were performed in the CIELAB color space, recording L* (lightness), a* (red-green), and b* (yellow-blue) coordinates. The instrument was calibrated using standard white and black reference tiles prior to measurements. All films with 16 mils thickness were measured in triplicate [21].

The total color difference (Δ*E*) between samples was calculated using the following Equation (1):

(1) $$∆E=\sqrt{{∆a^{*}}^{2}+{∆b^{*}}^{2}+∆{L^{*}}^{2}}$$

where ΔL*, Δa*, and Δb* represent the differences in respective color coordinates between samples. Additionally, the yellowness index (YI) was determined for all blend systems using Equation (2), following standard colorimetric procedures as reported by Pathare et al. (2013) [34].

(2) $$YI=\frac{142.86}{L^{*}}b^{*}$$

### 3.3. Mechanical Testing

Injection-molded specimens for mechanical testing were prepared from vacuum-dried (60 °C, 24 h) pellets of PHBV and its blends. Tensile properties were evaluated according to ASTM D638 using dumbbell-shaped specimens (165.0 × 12.4 × 3.2 mm) with a 115 mm grip distance. Tests were performed on an Instron 5985 tensiometer equipped with Bluehill v. 2.17 software (Instron Corp., Norwood, MA, USA) at room temperature with a crosshead speed of 50 mm/min. Flexural properties were determined following ASTM D790-15 using rectangular specimens (124.0 × 12.4 × 3.2 mm) under a 5 kN load cell capacity with a crosshead speed of 10 mm/min on the same instrument [35]. The reported values represent the average and standard deviation of at least ten specimens. Statistical analysis was performed using JMP Pro 16.0 (SAS Institute, Cary, NC, USA). Significant differences (*p* < 0.05) between PHBV and its blends were evaluated using one-way ANOVA followed by Tukey’s HSD test.

Dynamic mechanical analysis (DMA) was performed using a TA Instrument Q800 (New Castle, DE, USA) with tension film clamps. Specimens (16.0 mm × 7.0 mm × 0.6 mm) were equilibrated at −30 °C and heated to 160 °C at a rate of 5 °C/min, with a preload of 1 N and frequency of 1 Hz. The values presented are averages of four specimens.

### 3.4. Rheological Properties

The melt rheological behavior of PHBV and its blends was characterized using a Discovery HR-3 Rheometer (TA Instruments, DE, USA), equipped with 25 mm parallel plate geometry (ETC steel 113652) with a 1 mm gap setting at 175 °C. Prior to testing, samples were vacuum dried at 60 °C for 24 h. Dynamic oscillatory measurements were performed in triplicate to evaluate the viscoelastic properties. Initially, strain-sweep tests were conducted to determine the linear viscoelastic region (LVR). Frequency sweeps were then performed from 0.1 to 100 rad/s at a constant strain of 0.3%, which was within the identified LVR.

## 4. Results and Discussion

### 4.1. Effects of Coffee Oil Epoxide (COE) on the Structural Properties of PHBV/NR Blends

**Figure 2:**
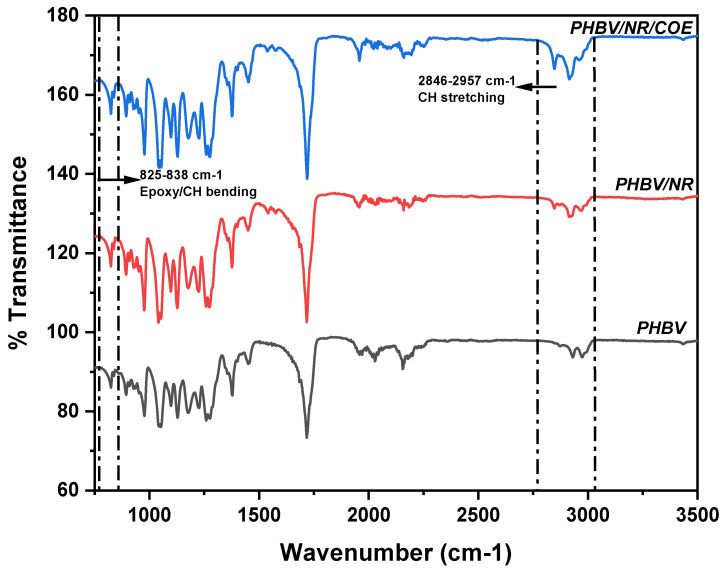
ATR-FTIR spectra of PHBV and its blends.

The ATR-FTIR spectra revealed characteristic absorption bands for PHBV and its blends (Figure 2). For neat NR, the characteristic peaks at 2957, 2915, and 2846 cm^−1^ were attributed to C-H stretching vibrations, specifically the asymmetric stretch in CH_3_ groups and the asymmetric and symmetric CH_2_ vibrations [36]. The peak at 1406 cm^−1^ corresponded to C-H bending of CH_3_ groups, while the band at 825 cm^−1^ was characteristic of =CH bending in the 1,4-distributed NR structure [36]. The FTIR spectrum of neat PHBV exhibited its characteristic crystalline C=O symmetric stretching in aliphatic esters at 1720 cm^−1^, along with C-O-C stretching vibration at 1100 cm^−1^. Additional peaks at 1450, 1376, and 826 cm^−1^ were assigned to various CH modes, including asymmetric deformation, symmetric deformation, and bending vibrations [37].

In the PHBV/NR/COE blends, several notable changes were observed compared to the neat components. The intensification of peaks at 2847 and 2920 cm^−1^, corresponding to CH2 stretching, could be attributed to the incorporation of COE. Interestingly, the peak at 2975 cm^−1^, associated with asymmetric CH stretching in the crystalline phase of PHBV, showed decreased intensity in the blends, suggesting reduced crystallinity. However, the peak at 2931 cm^−1^ exhibited higher intensity compared to neat PHBV due to the COE contribution.

During melt blending, the increased intensity of the 825 cm^−1^ peaks in PHBV/NR blends compared to neat PHBV indicates successful grafting between PHBV and NR chains [22]. This grafting occurs due to the high shear forces and elevated temperatures during extrusion, which generate free radicals through chain scission of both PHBV and NR molecules, enabling chemical bonding at their interface. However, the lower intensity of epoxy peaks (825–838 cm^−1^) in PHBV/NR/COE blends compared to PHBV/NR blends, while still being more intense than neat PHBV, suggests that during the extrusion process, epoxy groups from COE undergo ring-opening reactions and interact with both the terminal hydroxyl (-OH) groups of PHBV and the double bonds present in NR. This complex interaction results in crosslinking between the polymer chains, where some epoxy groups are consumed in the reaction, leading to the observed decrease in peak intensity. The FTIR spectrum of pure COE, included in the Supplementary Information (see Figure S1), supports the assignment of these epoxy-related absorption bands. These molecular-level interactions during melt blending create an interpenetrating network structure that significantly influences the final properties of the blends [38]. Beyond the primary structural changes, significant spectral modifications were observed in specific functional group regions. The enhanced intensity of the peak at 1406 cm^−1^ in PHBV/NR/COE blends, compared to both neat PHBV and PHBV/NR blends, suggests increased CH_3_ bending vibrations resulting from the complex interactions between blend components. More interestingly, the amide region exhibited distinct changes, with peaks at 1540 and 1575 cm^−1^, showing increased intensity in the blends. These peaks, corresponding to N-H stretch, C=O, and N-H bending of rubber proteins, provide evidence for the preservation of rubber protein structure during melt blending [22].

Chemometric analysis was performed on the FTIR spectral data to investigate molecular interactions and structural modifications in blends using Principal Component Analysis (PCA) and Partial Least Squares Discriminant Analysis (PLS-DA).

**Figure 3:**
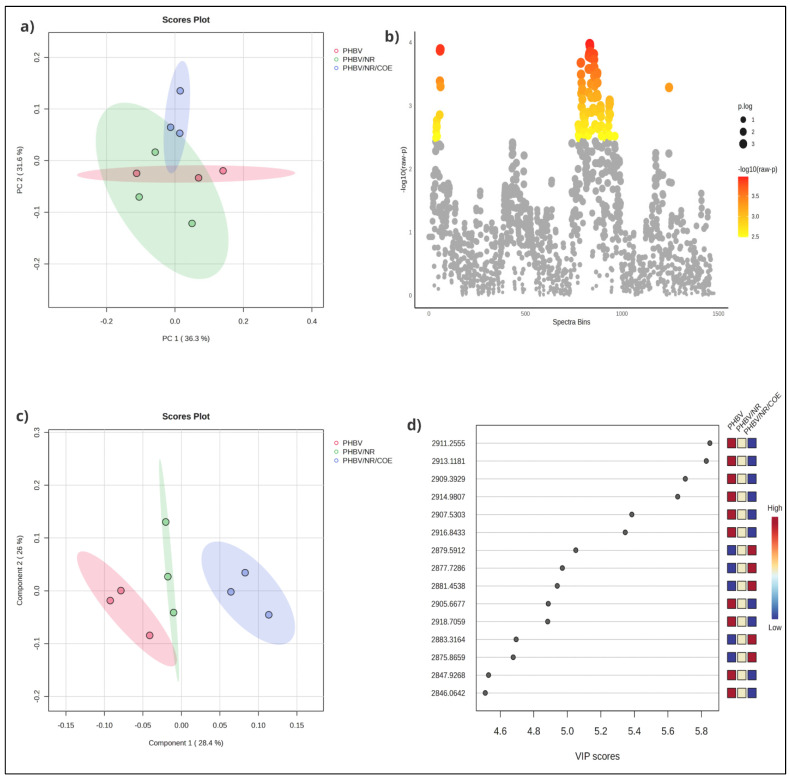
(**a**) 2D score plots of the PCA, showing sample clustering based on compositional similarities; (**b**) ANOVA results highlighting statistically significant spectral bins contributing to group differentiation; (**c**) PLS-DA score plot providing improved class separation among the formulations; and (**d**) VIP scores identifying critical spectral regions that most significantly contribute to blend discrimination.

PCA, as an unsupervised chemometric technique, was initially employed to evaluate the natural grouping tendencies within the dataset. Principal Components (PCs) are the transformed variables that capture the main patterns of variation in the data. After projecting the high-dimensional spectral data into two-dimensional space, distinct sample groupings emerged based on their temporal sampling points. The variance analysis revealed that PC1 accounted for 36.3% of the total variance, while PC2 explained an additional 31.6%, resulting in a cumulative explained variance of 67.9%. This distribution suggests that the majority of relevant spectral information was captured within the first two principal components (Figure 3a). However, the score plots demonstrate partial overlapping between PHBV and its blended formulations, indicating compositional similarities that precluded complete distinction between sample classes. To further elucidate the spectral differences between sample groups, Analysis of Variance (ANOVA) was performed, identifying 97 peaks that exhibited statistically significant differences between the formulations (Figure 3b). Of particular significance were the CH_2_ stretching vibrations at 2847 and 2914 cm^−1^, characteristic of COE incorporation, which displayed heightened intensity in PHBV/NR/COE blends compared to PHBV/NR and pristine PHBV samples. The ANOVA results also revealed significant variations in the PHBV crystallinity-associated peak at 2975 cm^−1^, which showed diminished intensity in the blended samples, providing statistical validation of reduced crystallinity upon blending. Additionally, Natural Rubber-specific peaks at 2914 and 2959 cm^−1^ demonstrated statistically significant variations in the blended formulations, confirming successful component integration and molecular-level interactions between blend constituents.

The effectiveness of the chemometric approach was further validated through Partial Least Squares Discriminant Analysis (PLS-DA), which showed improved class discrimination capabilities with PC1 and PC2 accounting for 28.4% and 26.0% of the variance, respectively (Figure 3c). The VIP analysis identified critical spectral regions that contributed most significantly to sample discrimination, particularly in the CH_2_ stretching region (Figure 3d). The highest VIP scores (>5.6), observed for peaks at 2911.2555, 2913.1181, and 2909.3929 cm^−1^, correlated strongly with the regions showing COE incorporation and blend formation. This statistically validates the significance of the observed increased intensity of CH_2_ stretching peaks in PHBV/NR/COE blends, as identified in the raw FTIR spectra. These findings align with the FTIR observations of intensified CH_2_ stretching peaks in PHBV/NR/COE blends. The intermediate Variable Importance in Projection (VIP) scores (5.0–5.4) for the peaks at 2879.5912, 2877.7286, and 2881.4538 cm^−1^ suggested alterations in PHBV crystallinity. Additionally, the lower VIP scores (4.6–4.8) at 2847.9268 and 2846.0642 cm^−1^ validated their importance in characterizing the blend interactions, supporting the observed spectral changes related to the epoxy ring-opening reactions between the blend components.

The correlation between chemometric analysis and spectral features provides strong evidence for the formation of an interpenetrating network structure through complex molecular interactions during melt blending. The statistical significance of spectral changes, particularly in regions identified by high VIP scores, confirms systematic relationships between blend composition, processing conditions, and resulting molecular structures. These findings offer valuable insights for optimizing blend formulations and processing conditions to achieve desired material properties.

### 4.2. Optical Characteristics of Blended Films with and Without Plasticizers

The optical and colorimetric properties of poly(3-hydroxybutyrate-co-3-hydroxyvalerate) (PHBV) blends were systematically investigated through CIELAB color space analysis (Table 2). Incorporation of natural rubber (NR) and coffee oil epoxide (COE) demonstrated significant modifications in transparency and color characteristics, quantified through *L*, *a*, b* parameters, and derived metrics.

The results indicate that incorporation of COE led to substantial enhancement in lightness (L), exhibiting a statistically significant increase of 7.73% (*p* < 0.05) from 80.33 ± 1.79 (neat PHBV) to 86.54 ± 0.356 (PHBV/NR/COE). The addition of NR showed minimal impact on color properties, with only a 1.01% decrease in L* value. However, the incorporation of COE led to a significant 7.73% increase in lightness, approaching values reported for PLA/PEG systems [39,40]. This enhancement in transparency suggests effective plasticization and improved optical clarity.

Chromatic coordinates demonstrated systematic variations across the blend compositions. The a* value exhibited a notable shift from 1.86 ± 0.67 (neat PHBV) to −0.15 ± 0.01 (PHBV/NR/COE), indicating a transition from red to neutral undertones. Concurrently, the b parameter decreased by 15.81% (from 30.42 ± 0.64 to 25.61 ± 0.04), reflecting reduced yellowness. These modifications correlate with the plasticizing effect of COE and its influence on polymer chain organization in the blend system.

Total color difference (Δ*E*) analysis revealed values consistently exceeding the threshold for very distinct color modification (Δ*E* > 3), with PHBV/NR/COE exhibiting a 5.26% increase compared to the control system [41]. This significant variation suggests substantial alterations in the optical characteristics of the polymer blend matrix.

The optical characteristics of PHBV blends were further characterized through detailed analysis of hue angle, chroma, and yellowness index parameters. The hue angle measurements revealed distinctive variations among the blend compositions. The PHBV/NR/COE ternary blend exhibited a 4.41% increase in hue angle (90.34 ± 0.02) compared to neat PHBV (86.52 ± 1.16), while the PHBV/NR binary blend showed a 3.25% decrease (83.71 ± 0.10). This contrasting behavior indicates that the incorporation of COE plays a crucial role in achieving more neutral coloration and suggesting improved miscibility in the ternary blend system.

Quantitative analysis of chroma (C*) values, which represent color intensity perception, exhibited a systematic decreasing trend across blend compositions. The initial incorporation of NR resulted in an 8.89% reduction from neat PHBV to PHBV/NR. Further addition of COE led to an additional decrease of 15.98% in PHBV/NR/COE. This progressive reduction in color intensity correlates with enhanced molecular mixing and improved blend compatibility between components.

The most significant modification was observed in the yellowness index (YI), demonstrating a substantial 21.9% reduction from pure PHBV (54.10) to PHBV/NR/COE (42.27). Neat PHBV exhibits a naturally yellowish tint, a characteristic also observed in PHBV films containing phenolic compounds [42]. The color changes induced by coffee oil epoxide (COE) and natural rubber (NR) can be attributed to their inherent coloration and potential interactions with the polymer matrix. Another contributing factor is thermal stability. Neat PHBV typically undergoes degradation at temperatures marginally above its melting point (~180 °C), leading to chain scission and formation of conjugated double bonds that contribute to yellowing. The incorporation of NR enhances thermal stability through efficient heat dissipation during processing, while COE’s plasticizing effect enables processing at reduced temperatures [43]. Additionally, COE provides further stabilization through its epoxide groups. This synergistic combination of NR and COE effectively mitigates thermal and oxidative degradation, resulting in superior color stability of the PHBV blends.

### 4.3. Impact of Plasticization on the Rheological Properties of PHBV and Its Blends

Oscillatory measurement is the most common dynamic study to determine the viscoelasticity and structural changes in polymeric blends. These dynamic studies, crucial for determining material processability, revealed that PHBV blends exhibit non-Newtonian behavior characterized by shear thinning [44]. At low frequencies, the blends demonstrate high viscosity due to polymer chain entanglements and strong intermolecular forces, typical of high-molecular-weight polymer melts. As shear rates increase, the anisotropic polymer chains with high molar mass disentangle and align along the shear direction, leading to reduced intermolecular interactions, increased free volume, and consequently lower viscosity.

**Figure 4:**
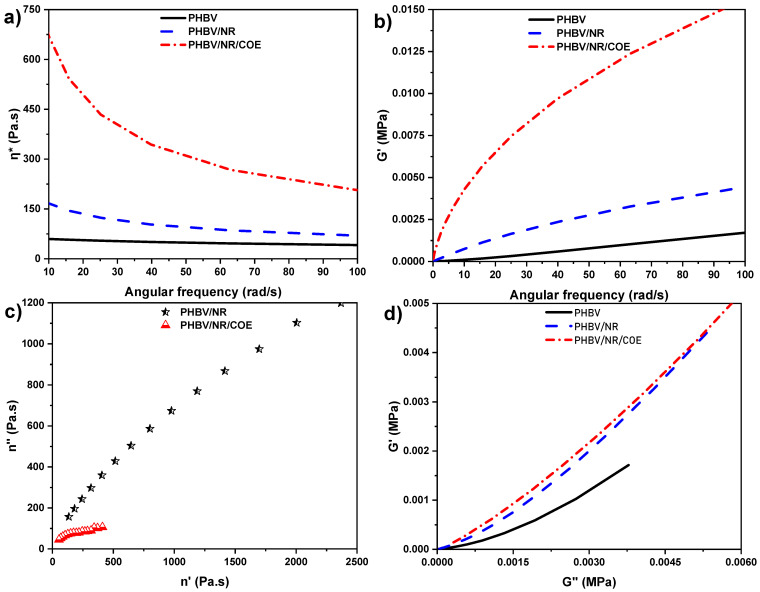
Rheological behavior of PHBV and its blends at 175 °C: (**a**) complex viscosity (η*) versus angular frequency showing shear-thinning behavior in all blends and anti-plasticization effect in COE blends; (**b**) storage modulus (G′) versus angular frequency demonstrating enhanced solid-like response with COE addition; (**c**) Cole–Cole plot (η″ versus η′) with semi-circular profiles indicating improved phase compatibility in PHBV/NR/COE blends compared to PHBV/NR; and (**d**) Han plot (G′ versus G″) showing increased linearity with COE incorporation, confirming reduced interfacial tension between polymer phases.

Interestingly, the complex viscosity (η*) of PHBV/NR/COE blends showed a more pronounced increase compared to neat PHBV and PHBV/NR blends (Figure 4a). This behavior can be attributed to an anti-plasticization effect at low COE concentrations (0.3%) in PHBV/NR blends. According to the fringed micelle theory, COE interacts with the amorphous regions of the polymer matrix, leading to slight increases in free volume and promoting crystallite formation [45]. This structural reorganization, combined with COE’s high viscosity at the processing temperature (175 °C, which is above PHBV’s melting point of ~170 °C but below COE’s melting point), contributes to increased rigidity and complex viscosity. Similar rheological behavior was reported by Li and Huneault. (2011) in sorbitol-TPS systems, where increased melt viscosity correlates with enhanced modulus and tensile properties [46].

Dynamic rheological measurements indicate that PHBV/NR blends exhibit predominantly liquid-like characteristics, enhanced by COE addition. The frequency dependence of storage (G′) (Figure 4b) and loss (G″) moduli, where G′ > G″, indicates a dominant elastic response. The higher G′ values correspond to longer relaxation times in the COE blends. While COE incorporation increased G′ values, suggesting enhanced solid-like behavior, PHBV/NR/COE demonstrated higher elasticity compared to PHBV/NR at equivalent G′ values. As shown in Figure 4, neat PHBV showed liquid-like behavior, whereas PHBV blends exhibited predominant solid-like behavior at low frequencies.

For qualitative evaluation of blend miscibility, Cole–Cole diagrams were plotted (Figure 4c). While Cole–Cole plots are widespread in the field of dielectric materials, they have not been extensively used in rheology [47]. The Cole–Cole plot, depicting the imaginary viscosity (η″) versus the real viscosity (η′), serves as a valuable tool for investigating the morphology and compatibility within polymer blends. In this study, the Cole–Cole plot was employed to assess the compatibility between PHBV, NR, and COE. The viscosity components η′ and η″ were derived from storage modulus (G′) and loss modulus (G″) using the relationship η′ = G′/ω and η″ = G″/ω (ω being the angular frequency) [48].

The parameters h, k, and H are critical indicators of polymeric structure and microscopic defects at the monomer level. Studies by Alberola and Bergeret. (1994) demonstrated that stronger interfacial interactions in polymer-filler systems correspond to decreased h values, indicating reduced chain mobility and flexibility [49]. Perez et al. developed a model expressing the complex shear modulus (G*) as a function of angular frequency (ω), relaxed modulus (*G_c_*), unrelaxed modulus (*G_L_*), and molecular relaxation time (*τ_mr_*), with additional parameters (h, k, and H) obtained from Cole–Cole plots (Equation (3)) [50].

(3) $$G^{*}=G^{\prime }+iG\prime \prime =G_{C}+\frac{G_{L}-G_{C}}{1+H{\left(i\omega \tau_{mr}\right)}^{-h}+{\left(i\omega \tau_{mr}\right)}^{-k}}\;$$

In the present study, conducting measurements at a single temperature (175 °C) precluded determination of *G_c_* and *G_L_* parameters [51]. Therefore, the Cole–Cole curves were fitted with a fourth-order polynomial to calculate the h parameter (Equation (4)), which provides valuable insights into chain branching and crosslinking occurring during extrusion.

(4) $$h=2\pi \arctan\;{\left(\frac{dG\prime \prime }{dG^{\prime }}\right)}_{G^{\prime }\;\to 0}\;$$

The Cole–Cole plots (Figure 4c) revealed characteristic features indicative of blend compatibility. A smooth, semi-circular curve indicates homogeneous phase distribution and effective molecular-level interactions between blend components in the molten state. Deviations from this ideal semi-circular shape suggests phase segregation, arising from polymer incompatibility where components separate into distinct domains rather than forming a uniform blend [52]. In contrast, the PHBV/NR/COE blends exhibited Cole–Cole plots that more closely resembled a semi-circular profile, indicating enhanced phase homogeneity compared to the PHBV/NR blends, which displayed more pronounced deviations from the ideal semi-circular shape. Analysis of the Cole–Cole plots demonstrated that PHBV/NR/COE blends closely resembled a semi-circular profile, indicating enhanced phase homogeneity compared to PHBV/NR blends, which showed more pronounced deviations from ideal semi-circular shape. These observations align with previous findings reported for PLA-clay systems [53]. However, it should be noted that Cole–Cole analysis alone is insufficient to fully characterize blend miscibility, necessitating additional rheological evidence to support these observations.

The h parameter values, determined from schematic diagrams in Figure 4d and Equation (4), were found to be 8.89 and 6.15 for PHBV/NR and PHBV/NR/COE blends, respectively. The observed low standard deviations (Table 3) demonstrated high measurement repeatability. The h values located within the range of [6,7,8,9], indicating characteristic branched polymer behavior, where lower values corresponded to increased branching and crosslink density [54]. PHBV/NR/COE blends exhibited the lowest h value, suggesting enhanced crosslinking compared to PHBV/NR blends. This observation correlated with the higher storage modulus (E′) values observed for PHBV/NR/COE blends, as discussed in the subsequent section. The increased crosslink density in PHBV/NR/COE blends resulted in restricted molecular mobility during rheological measurements, which was consistent with the observed dynamic mechanical behavior.

To further corroborate the miscibility findings from the Cole–Cole plots, Han plots were constructed for the PHBV/NR blends with and without coffee oil epoxide (COE). These plots, established by Han and Chuang (1985) [55], utilize the relationship between storage modulus (G′) and loss modulus (G″) in the low-frequency regime, where miscible blends exhibit a straight-line trend on a log–log plot, while deviations from linearity indicate phase separation.

As shown in Figure 4d, the Han plots reveal distinct rheological behaviors between the PHBV/NR and PHBV/NR/COE systems. The PHBV/NR blend demonstrates significant deviation from linearity at low frequencies, suggesting immiscibility between the phases. The limited interfacial adhesion between PHBV and NR phases likely resulted from polymer crosslinking—primarily, PHBV grafts onto NR at low loadings and NR-NR crosslinks at higher concentrations—due to their inherent incompatibility arising from polarity differences [43]. This finding is further supported by the slope variation in Han’s plot. Upon addition of COE, the blend exhibits a more linear profile with a different slope compared to the PHBV/NR blends. This linearity suggests that COE’s plasticizing effect improves chain mobility while simultaneously reducing interfacial tension between PHBV and NR phases. The resulting linear profile in the Han plot indicates enhanced phase mixing, which aligns with the observed blend morphology from SEM analysis [24].

### 4.4. Mechanical Properties of the Blends

**Figure 5:**
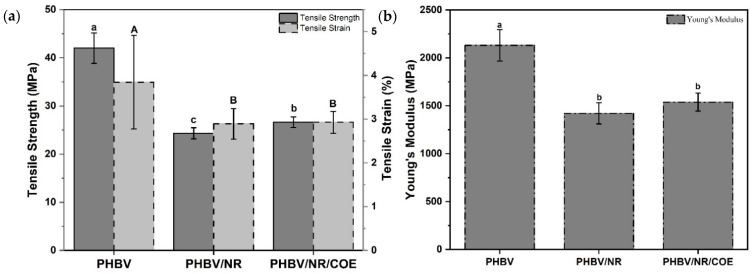
Mechanical properties of PHBV and its blends: (**a**) tensile strength and strain and (**b**) Young’s modulus. Different letters indicate significant differences between samples (*p* < 0.05). [Lower case letters and capital letters are being compared separately].

The tensile properties of PHBV and its blends with NR and COE were systematically investigated, and the results are presented in Figure 5a. The incorporation of NR and COE significantly influenced the mechanical behavior of PHBV. Pure PHBV exhibited a tensile strength of 42.02 ± 3.14 MPa, which decreased substantially to 24.33 ± 1.15 MPa upon NR addition, representing a 42.1% reduction. This decrease can be attributed to the softening effect of NR in the PHBV matrix. The Young’s modulus followed a similar trend, decreasing from 2130.92 ± 163.99 MPa to 1421.28 ± 110.10 MPa with NR addition, showing a 33.3% reduction (*p* < 0.05). This behavior indicates enhanced flexibility in the blend system due to the elastomeric nature of NR. This aligns with observations reported in other studies on PHBV/NR latex blends [56].

Interestingly, the incorporation of COE into the PHBV/NR blend resulted in a slight increase in tensile strength to 26.63 ± 1.10 MPa, representing a modest improvement of 9.4% compared to PHBV/NR. Similarly, the Young’s modulus showed an unexpected increase to 1538.79 ± 94.13 MPa, an 8.3% improvement over PHBV/NR (Figure 5b). This behavior contradicts typical plasticization effects, where the addition of plasticizers generally results in a decrease in mechanical properties. As explained by Platzer et al. (1982) plasticization at the molecular level typically involves the weakening of secondary bonds between polymer chains, increasing intermolecular space (free volume), which should lead to reduced mechanical properties [57,58]. However, the unexpected improvement in mechanical properties suggests that COE may function not only as a plasticizer but potentially as a compatibilizer between PHBV and NR phases.

Young’s modulus measurements followed a similar trend, with pure PHBV showing the highest value of 2130.921 ± 163.99 MPa. The addition of NR resulted in a 33.3% reduction to 1421.283 ± 110.1 MPa, while subsequent COE incorporation slightly increased the modulus to 1538.793 ± 94.13 MPa. This improvement in both tensile strength and Young’s modulus with COE addition suggests its potential role as a compatibilizer between PHBV and NR phases. The coefficient of variation for Young’s modulus remained relatively stable across all compositions, ranging from 7.7% to 6.12%, indicating consistent measurement reliability.

An unexpected trend was observed in the tensile strain behavior of the blends. Pure PHBV demonstrated the highest strain at break (%) of 3.844 ± 1.07, which decreased to 2.894 ± 0.35 with NR addition. The incorporation of COE resulted in minimal change to the strain (2.929 ± 0.25). This behavior contradicts the typical plasticization effect expected from COE addition, which usually enhances material ductility. Choi and Park. (2004) reported similar findings in their investigation of PHB systems, where plasticizer concentrations below 10 wt% resulted in minimal improvements or even decreases in elongation at break [59]. In our study, the reduction in strain could be attributed to several factors, including potential poor interfacial adhesion between phases, formation of a more brittle structure due to phase separation, or insufficient COE concentration to significantly impact chain mobility. Notably, the coefficient of variation for strain measurements showed substantial improvement from 27.75% for pure PHBV to 8.49% for PHBV/NR/COE, suggesting more uniform deformation behavior in the modified blends.

**Figure 6:**
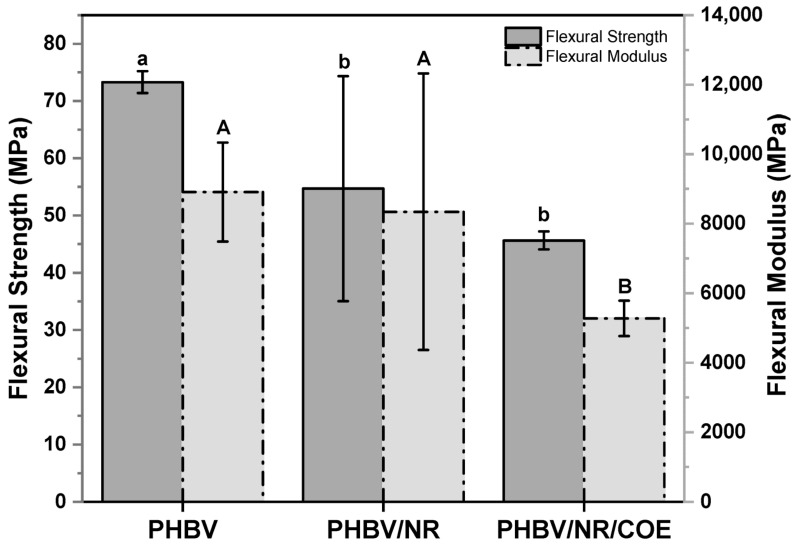
Flexural strength and modulus of PHBV and its blends. Error bars represent standard deviation (SD) based on at least ten replicates. The larger error bars for PHBV/NR are due to blend heterogeneity and phase separation. Different letters indicate significant differences between samples (*p* < 0.05). [Lower case letters correspond to statistical comparisons of flexural strength values, while capital letters denote statistical differences among flexural modulus measurements].

Flexural testing revealed complementary insights into the mechanical behavior (Figure 6). Pure PHBV exhibited the highest flexural strength of 73.31 ± 1.91 MPa and flexural modulus of 8909.93 ± 1423.51 MPa, demonstrating its inherent rigid and brittle nature. Upon incorporation of NR into PHBV, a notable decrease in flexural strength to 54.69 ± 19.66 MPa was observed, representing a 25.4% reduction from pure PHBV. This blend showed a relatively modest decrease in flexural modulus of 6.4%, reaching 8341.80 ± 3978.63 MPa. However, the high standard deviation in the PHBV/NR blend suggests potential issues with blend homogeneity or phase separation between the components.

The addition of COE to the PHBV/NR blend resulted in further modifications to the mechanical properties. The flexural strength decreased to 45.65 ± 1.58 MPa, marking a reduction of 16.5% compared to PHBV/NR blends. More dramatically, the flexural modulus showed a significant decrease of 36.8%, dropping to 5274.27 ± 510.42 MPa. The lower standard deviations in the PHBV/NR/COE blend indicate improved homogeneity compared to the PHBV/NR blend. These results suggest that COE acts as an effective plasticizer in the system, enhancing the material’s flexibility while reducing its structural rigidity. The sequential decrease in both flexural strength and modulus with the addition of NR and COE demonstrates the transformation of PHBV from a rigid material to a more flexible composite, which could be advantageous for applications requiring enhanced ductility rather than high strength and stiffness. The divergent behavior is attributed to the distinct nature of loading conditions and the dual role of COE in the blend system. Under flexural loading, the material experienced both compression and tension simultaneously, making the plasticization effect more prominent. However, under pure tensile loading, it appears to act as a compatibilizer, improving the interfacial adhesion between the PHBV and NR phases.

The results indicate that coffee oil epoxide influenced the mechanical properties through plasticization, as well as modifications to the material’s morphology and interfaces within the blend system. The material’s contrasting tensile and flexural behavior suggests its response depends on the applied stress and resulting molecular rearrangements.

### 4.5. Dynamic Mechanical Analysis of the PHBV and Its Blends

**Figure 7:**
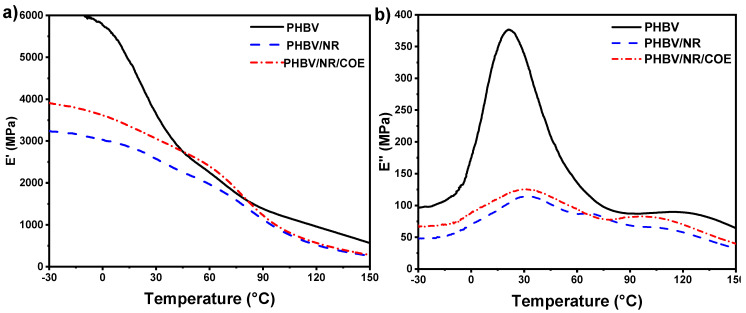
(**a**) Storage modulus vs. temperature and (**b**) storage modulus vs. temperature of PHBV and its blends.

The viscoelastic properties of PHBV and its blends were investigated using Dynamic Mechanical Analysis as illustrated in Figure 7. Significant variations in storage modulus (E′), loss modulus (E″), and damping factor (tan $\delta =\frac{E^{\prime \prime }}{E^{\prime }}$) were observed as functions of temperature across all formulations. The glass transition (T_g_) events were discernible through characteristic declines in the storage modulus concurrent with corresponding peaks in the loss modulus and tangent delta curves.

The E′, which indicates material stiffness and correlates with Young’s modulus, demonstrated that neat PHBV exhibited the highest initial E′ values (6.0–5.7 GPa) between −30 and 2 °C, reflecting its inherent brittleness (Figure 7a). A pronounced reduction in E′ of 2.5 GPa was observed between 2 and 68 °C, corresponding to the glass transition region where molecular mobility increases. The E″ curve for PHBV showed a peak maximum at approximately 29 °C, confirming this transition temperature (Figure 7b).

In the temperature range from −30 to 150 °C, PHBV/NR blends demonstrated consistently lower E′ values than neat PHBV, indicating enhanced elasticity due to NR incorporation [60]. The addition of NR resulted in a characteristic three-stage modulus reduction: an initial gradual decrease from −25 to 15 °C, followed by a significant decline between 15 and 55 °C, and a final drop above 55 °C. This behavior, coupled with two distinct maxima in both E″ and tan δ curves, provided evidence of phase separation and α’ relaxation in the amorphous–crystalline interphase, confirming the immiscibility between PHBV and NR phases. Similar behavior was recently reported for PHBV plasticized with an oligomeric polyester based on lactic acid, adipic acid, and 1,2-propanediol (PLAP) [61].

The incorporation of COE significantly modified the blend behavior, as evidenced by the transformation to a single relaxation transition, suggesting improved compatibility with COE incorporation. Interestingly, PHBV/NR/COE blends demonstrated higher storage modulus values (3.9 GPa) compared to PHBV/NR systems (3.2 GPa), contradicting conventional plasticization mechanisms. A plasticizer is known to increase the segmental motion of the polymer backbone and decrease the storage modulus [62]. These findings support the tensile data that COE’s role extends beyond simple plasticization, potentially facilitating enhanced intermolecular interactions and contributing to the formation of a more cohesive network structure, thereby altering the overall mechanical response of the blend. The observed modulus behavior was attributed to partial miscibility and the limited plasticizing efficiency of COE at 0.3 wt%, potentially indicating saturation effects leading to phase-separated microdomains [27].

**Figure 8:**
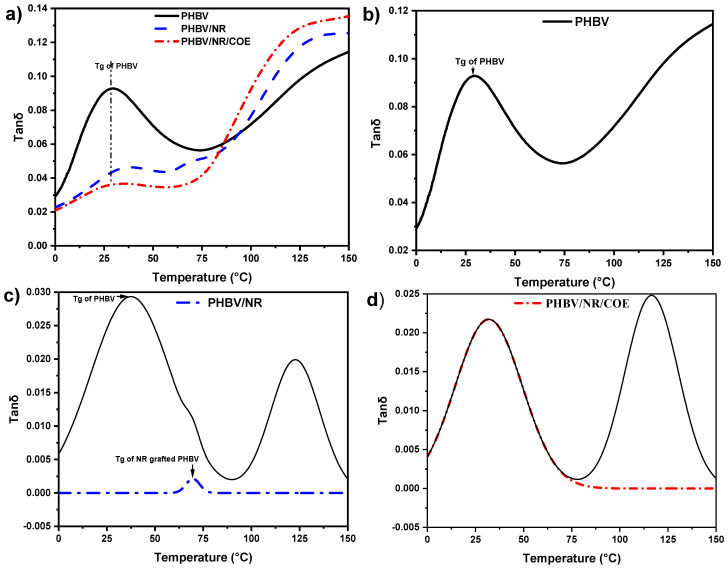
(**a**) Tan δ vs. *T* curves showing distinct glass transition behavior across formulations; (**b**) Gaussians curves for PHBV demonstrating homogeneous single-phase relaxation; (**c**) Gaussians curves (blue and red lines) and the sum of the Gaussians (black line) for PHBV/NR blends revealing bimodal distribution; and (**d**) Gaussian deconvolution showing individual peaks (red line) and original tan δ curve (black line) for PHBV/NR/COE blend exhibiting a single broadened peak, confirming improved miscibility and enhanced interfacial interactions upon COE incorporation.

Analysis of tan δ curves revealed distinct transitions in the blend systems (Figure 8a). PHBV/NR blends showed two α-relaxation peaks, confirming thermodynamic immiscibility. However, PHBV/NR/COE blends exhibited a single relaxation peak at ~32 °C, compared to neat PHBV’s T_g_ at ~29 °C. This single transition suggested enhanced compatibility and reduced phase separation with COE addition. The deviation between these T_g_ values and those obtained from DSC measurements, as previously reported, can be attributed to differences in T_g_ determination methods, where DMA assesses dynamic thermomechanical properties, while DSC measures thermal transitions. Neat PHBV displayed the highest tan δ value, indicating more viscous behavior compared to the elastic-dominated blends. In contrast, PHBV/NR and PHBV/NR/COE blends exhibited broader and lower damping peaks, consistent with enhanced chain mobility and increased free volume. The observed reduction in the softening point temperature with NR and COE incorporation provided further evidence of enhanced chain mobility. The reduced tan δ values across all blends align with DSC results, confirming that NR and COE improve polymer chain motion while reducing stiffness.

Further analysis through tan δ deconvolution provided critical insights into the phase behavior and molecular dynamics of PHBV-based blends. Neat PHBV demonstrated a single Gaussian peak (Figure 8b), indicative of homogeneous molecular relaxation processes characteristic of single-phase systems. In contrast, PHBV/NR blends exhibited two distinct peaks (Figure 8c), confirming phase separation typically observed in immiscible polymer systems. ORIGIN deconvolution analysis identified an additional peak, suggesting the presence of a molecular network with enhanced mobility compared to the primary matrix. The Gaussian function was found to provide the best fit for the experimental tan δ values of PHBV blends. While some degree of speculation and optimization was involved in selecting the appropriate deconvolution model, multiple curve-fitting approaches were systematically tested. The final selection was based on using the fewest number of curves necessary to achieve a statistically valid fit.

The deconvolution analysis revealed two distinct peaks in PHBV/NR blends, indicating different molecular mobility regions. The lower temperature peak can be attributed to the mobile amorphous phase, while the higher temperature peak suggests a more restricted molecular mobility region. Similar behavior has been reported in immiscible polymer blends by Qiu et al. (2013), who demonstrated that such dual relaxation behavior in polymer blends often indicates distinct mobility regions arising from phase separation and interfacial interactions [63]. The presence of dual glass transitions in PHBV/NR blends, with T_g_ at 37 °C (PHBV) and 69 °C (NR-grafted PHBV), respectively, confirms phase separation with minimal interfacial interaction. This behavior is typical of immiscible polymer blends, where components maintain their individual thermal transitions due to limited molecular mixing at the interface. The sharp interface and distinct phase domains further support the immiscible nature of the PHBV/NR system.

Interestingly, upon incorporation of COE, the blend transitions to a single, broader tan δ peak, indicating enhanced molecular mixing and partial miscibility (Figure 8d). This observation aligns with our previous morphological findings, where COE addition resulted in reduced NR droplet size. The broadening of the tan δ peak suggests a wider distribution of relaxation times, characteristic of improved interfacial interaction and chain mobility.

The overlapping transitions in tan δ and E′ curves suggest the absence of sharp phase boundaries, instead pointing to a gradual mobility gradient between phases. This behavior, previously reported by Barbosa et al. (2021), indicates progressive changes in chain mobility from crystalline to mobile amorphous regions [61]. The broader tan δ peak in PHBV/NR/COE blends, compared to PHBV/NR, suggests reduced phase separation and improved interfacial interaction, consistent with the compatibilizing effect of COE [27]. These findings demonstrate that COE effectively modifies the phase behavior of PHBV/NR blends, transitioning from a clearly immiscible system to one exhibiting partial miscibility, as evidenced by both thermal and morphological characteristics. The correlation between tan δ peak characteristics and blend morphology provides valuable insights into the structure–property relationships in these complex polymer systems.

### 4.6. Summary of Key Findings

Table 4 summarizes the comprehensive characterization of PHBV/NR and PHBV/NR/COE blends, highlighting the significant changes resulting from coffee oil epoxide (COE) incorporation. Various analytical techniques were employed to evaluate structural, mechanical, optical, barrier, crystalline, and morphological properties of the blends. The results collectively demonstrate the effects of COE addition on the properties of the PHBV/NR blend.

## 5. Conclusions

This study comprehensively investigated the effectiveness of coffee oil epoxide as a bio-based plasticizer for blends of polyhydroxybutyrate-co-valerate and natural rubber. The incorporation of coffee oil epoxide was validated through various analytical methods, including FTIR-chemometric analysis that revealed characteristic structural changes and enhanced CH_2_ stretching intensities. Furthermore, rheological characterization provided substantial evidence of improved phase compatibility, as indicated by a significant reduction in the relaxation time distribution parameter and a transition towards near-linear Han plot profiles. The findings conclusively demonstrate the dual role of COE as both a plasticizer and a compatibilizer, as evidenced by the mechanical property evaluations. While COE displayed the typical plasticization behavior under flexural loading, it simultaneously enhanced the tensile properties, confirming its compatibilizing effect at the PHBV/NR interface. Further corroboration came from the dynamic mechanical analysis, where the transformation from bimodal transitions to a single, broader relaxation provided compelling evidence of improved miscibility and interfacial interactions. The findings further indicate that the improvements in optical properties, such as increased lightness and decreased yellowness index induced by COE, accentuate its potential to enhance the commercial viability of the material for packaging applications. Collectively, these results establish COE as an effective and sustainable additive that can enhance the processability and performance of biodegradable PHBV-based materials, while also contributing to the principles of the circular economy through the valorization of food waste.

Future research should focus on comprehensively evaluating the migration potential of COE in food packaging applications, assessing the impact of COE on the biodegradation of PHBV/NR blends, and investigating the aging characteristics of these materials under various environmental conditions. Additionally, impact resistance characterization through Izod and Charpy pendulum tests according to ASTM D256 would provide crucial insights into the material’s energy absorption capacity and failure mechanisms under high-speed loading conditions—properties essential for packaging materials that must withstand dropping and handling during transportation [64]. These investigations would establish essential performance parameters necessary for commercial translation of these sustainable materials [65].

## Figures and Tables

**Table 1:** Barrel and Die Temperature Profile During Melt Processing.

Zone	Barrel Temperature (°C)
T_1_	180
T_2_	180
T_3_	178
T_4_	178
T_5_	175
T_6_	175
T_7_	167
T_8_	165
T_9_	165
Die	162

**Table 2:** Optical properties of PHBV and its blends.

	Colorimeter						
Sample	L*	a*	b*	Δ*E*	hue	Chroma	Yellowness Index (YI)
PHBV	80.33 ± 1.79 ^b^	1.86 ± 0.67 ^b^	30.42 ± 0.64 ^a^	85.87 ± 1.45 ^b^	86.52 ± 1.16 ^b^	30.48 ± 0.68 ^a^	54.10 ± 0.52 ^a^
PHBV/NR	79.52 ± 0.33 ^b^	3.04 ± 0.04 ^a^	27.53 ± 0.36 ^b^	84.14 ± 0.27 ^c^	83.71 ± 0.10 ^c^	27.77 ± 0.38 ^b^	49.46 ± 0.71 ^b^
PHBV/NR/COE	86.54 ± 0.36 ^a^	−0.15 ± 0.01 ^c^	25.61 ± 0.04 ^c^	90.39 ± 0.04 ^a^	90.34 ± 0.02 ^a^	25.61 ± 0.04 ^c^	42.27 ± 0.63 ^c^

Note: Values with the same letters are not statistically significant at *p* ≤ 0.05. The asterisk in L*, a*, and b* is part of standard CIELAB notation and does not indicate statistical significance.

**Table 3:** Cole–Cole and Han Plot Parameters for PHBV and its Blends.

Sample	h Parameter	Han Plot Slope	R^2^ Value
PHBV	-	0.42 ± 0.02	0.95695
PHBV/NR	8.89	0.80 ± 0.03	0.97978
PHBV/NR/COE	6.15	1.12 ± 0.04	0.98421

**Table 4:** Comparative Characterization of PHBV/NR and PHBV/NR/COE Blends.

Analytical Technique	Parameter Measured	PHBV/NR	PHBV/NR/COE	% Change	Significance
**Structural Properties**	CH₂ stretching intensity (2847, 2920 cm^−1^)	Low	High	67.9% (PCA variance)	Confirms successful COE incorporation
**Mechanical Properties**	Cole–Cole Plot: Relaxation time distribution parameter (h)	8.89	6.15	~30.8%	Enhanced crosslinking density
	Han plot: Slope linearity	Non-linear	Near linear	—	Reduced interfacial tension
	Tensile strength (MPa)	24.33 ± 1.15	26.63 ± 1.10	~9.4%	Enhanced interfacial adhesion
	Young’s modulus (MPa)	1421.283 ± 110.1	1538.793 ± 94.13	~8.3%	Compatibilizing effect
	**Flexural strength (MPa)**	**54.69 ± 19.66 MPa**	**45.65 ± 1.58 MPa**	**~16.5%**	**Plasticization effect**
	**Flexural modulus (MPa)**	**8341.80 ± 3978.63**	**5274.27 ± 510.42**	**~36.8%**	**Enhanced chain mobility**
	DMA: Glass transition temperatures (°C)	37 and 69 °C	~32 °C	—	Improved phase miscibility
	tan δ peak profile	Bimodal	Single broad	—	Enhanced interfacial interactions
**Optical Properties**	Lightness (L*)	79.52 ± 0.33	86.54 ± 0.36	~8.83%	Improved visual appearance
	Yellowness Index	49.46 ± 0.71	42.27 ± 0.63	~14.54%	Enhanced thermal stability
**Barrier** ^1^ **Properties**	**WVTR (g/m^2^/day)**	**4.05 ± 0.54**	**1.55 ± 0.20**	**~61.7%**	**Enhanced barrier properties**
	**OTR (cc/m^2^/day)**	**2025.29 ± 35.69**	**21.51 ± 1.68**	**~98.9%**	**Significantly improved barrier**
	Water uptake (%)	3.54	1.36	~61.6%	Reduced hydrophilicity
	**Contact angle (°)**	**69.47**	**71.12**	**~2.3%**	**Increased surface hydrophobicity**
**Crystalline Properties** ^1^	Crystallinity (%)	63.52	68.85	~8.4%	Enhanced crystalline structure
	Crystal size (nm)	22.67	20.32	~10.4%	More numerous, smaller crystallites
	d-spacing (020) plane (Å)	5.86	6.51	~11.1%	More perfect crystals with improved long-range order
**Morphological Properties** ^1^ **(SEM)**	Phase dispersion	Distinct phases	Homogeneous	—	Elimination of microdroplets
**Processability** ^1^	Extrusion homogeneity	Inconsistent	Good	—	Improved industrial processability

Note: ^1^ Data adapted from our previously published paper [24]. Bold text indicates parameters with significant improvements in the PHBV/NR/COE blend compared to PHBV/NR blend.

## Data Availability

The data supporting the findings of this study are presented within the article and available on request from the corresponding author.

## Supplementary Materials

The following supporting information can be downloaded at: https://www.mdpi.com/article/10.3390/polym17101324/s1, Figure S1: FTIR spectrum of pure coffee oil epoxide (COE); Table S1: Assignment of main absorption bands in the FTIR spectrum of COE.
