# Supplementary material for: Rheological Behavior and Mechanical Performance of Poly(3-hydroxybutyrate-co-3-hydroxyvalerate)/Natural Rubber Blends Modified with Coffee Oil Epoxide for Sustainable Packaging Applications
Source: Polymers (Basel). 2025 May 13;17(10):1324. doi: 10.3390/polym17101324 (PMC12115077; doi:10.3390/polym17101324)
Supplement: Supplementary file 1 [file polymers-17-01324-s001.zip › polymers-3629052-supplementary.pdf]

Rheological Behavior and Mechanical Performance of Poly(3-hydroxybutyrate-co-3-hydroxyvalerate)/Natural Rubber Blends Modified with Coffee Oil Epoxide for Sustainable Packaging Applications

Rinky Ghosh[1], Xiaoying Zhao[2], Yael Vodovotz[1*]

[1] Department of Food Science and Technology, The Ohio State University, 2015 Fyffe Road, Columbus, OH, 43210, USA

[2] School of Light Industry Science and Engineering, Beijing Technology and Business University, No. 33 Fucheng Road, Beijing, 100048, China

*Correspondence: vodovotz.1@osu.edu, Tel.: +01- 614-247-7696

Supplementary Information

4.1 Effects of Coffee Oil Epoxide (COE) on the Structural Properties of PHBV/NR Blends

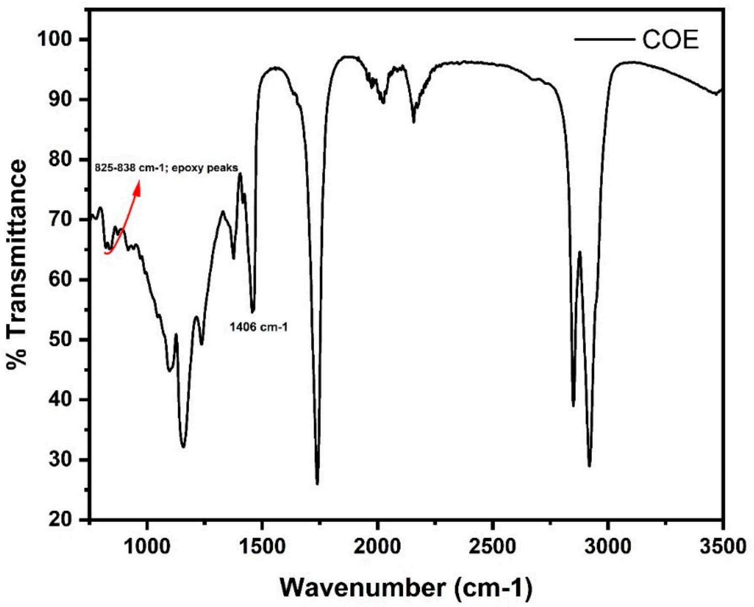

Figure S1. FTIR spectrum of pure coffee oil epoxide (COE).

FTIR spectrum of pure coffee oil epoxide (COE) showing characteristic epoxy absorption peaks in the region 825–838 cm^{-1}, confirming the presence of epoxide functional groups. The band at 1406 cm^{-1} corresponds to CH_3 bending vibrations. This spectrum provides reference data for interpreting the chemical interactions discussed in Section 4.1 of the main manuscript regarding the ring-opening reactions between COE and PHBV/NR blends.

Table S1: Assignment of main absorption bands in the FTIR spectrum of COE

Wavenumber (cm^{-1})	Assignment
825-838	C-O-C stretching of epoxide ring
1406	CH_3 bending vibrations
2847-2957	C-H stretching vibrations
